# Case Report: A rare case of serous cystadenocarcinoma of the ovary with a benign teratoma in the other ovary

**DOI:** 10.3389/fmed.2026.1729956

**Published:** 2026-02-06

**Authors:** Abhishek Ghose Biswas, Kishore Hiwale

**Affiliations:** Department of Pathology, Jawaharlal Nehru Medical College, Datta Meghe Institute of Higher Education and Research, Wardha, Maharashtra, India

**Keywords:** bilateral ovarian tumors, histopathology, mature cystic teratoma, ovarian neoplasms, serous cystadenocarcinoma

## Abstract

Synchronous ovarian tumors of different histogenesis are rare. High-grade serous carcinoma (HGSC) is an aggressive epithelial malignancy that primarily affects postmenopausal women, whereas mature cystic teratoma is a benign germ cell tumor usually seen in younger patients. Their occurrence in contralateral ovaries poses significant diagnostic challenges. A 57-year-old postmenopausal woman presented with abdominal distension, discomfort, and ascites. Imaging revealed a solid-cystic right ovarian mass with peritoneal involvement and a left adnexal lesion showing fat–fluid levels suggestive of a dermoid cyst. CA-125 was markedly elevated, and ascitic cytology confirmed malignant epithelial cells. She underwent a hysterectomy with bilateral salpingo-oophorectomy and omentectomy. Histopathology showed high-grade serous carcinoma in the right ovary with tubal and cervical stromal invasion, while the left ovary contained a benign mature cystic teratoma. Immunohistochemistry supported Müllerian origin (WT-1+, PAX8+, p53 mutant pattern, and high Ki-67). The tumor was staged as FIGO IIIC. Postoperative platinum-based chemotherapy resulted in significant clinical improvement, CA-125 reduction, and no recurrence at 9 months. This synchronous presentation underscores the importance of correlating imaging, cytology, extensive sampling, and immunohistochemistry to distinguish independent tumors from bilateral carcinoma. Accurate diagnosis enables appropriate oncologic management while avoiding overtreatment of benign lesions.

## Introduction

Ovarian neoplasms comprise a diverse spectrum of epithelial, germ cell, and sex cord–stromal tumors, each with distinct origins, clinical presentations, and biological behaviors. High-grade serous carcinoma (HGSC) is the most prevalent and aggressive subtype of epithelial ovarian cancer, typically affecting postmenopausal women and presenting at advanced stages due to vague or non-specific symptoms. It characteristically demonstrates complex papillary structures, marked cytologic atypia, stromal invasion, psammoma bodies, and a well-established association with Müllerian epithelium, particularly the fimbrial end of the fallopian tube (1). Treatment requires complete cytoreductive surgery and platinum-based chemotherapy owing to its high metastatic potential and rapid peritoneal dissemination.

In contrast, mature cystic teratoma, a benign germ cell tumor composed of tissues derived from all three germ layers, is most commonly encountered in younger women and often discovered incidentally unless complicated by torsion or rupture (2). Although both tumors are frequently observed independently in clinical practice, their **synchronous occurrence in contralateral ovaries** is extremely rare. Unlike collision tumors—where two distinct neoplasms develop within the same ovary without intermixing—contralateral synchronous tumors arise independently, and their coexistence can obscure preoperative diagnostic impressions and influence staging and management (3).

From a developmental perspective, the distinct histogenesis of these tumors supports independent tumorigenic pathways: HGSC arises from Müllerian epithelium, whereas teratomas originate from totipotent germ cells capable of differentiating into ectodermal, mesodermal, and endodermal components (4). This distinction emphasizes the importance of extensive tissue sampling, as HGSC may involve both ovaries and teratomas may rarely undergo malignant transformation. Misinterpretation of bilateral ovarian masses can lead to inappropriate staging, either underestimating carcinoma extent or overtreating a benign lesion.

The present case involves a 57-year-old postmenopausal woman with no significant family history of ovarian or breast malignancy, who presented with progressive abdominal discomfort and bilateral adnexal masses. Her demographic profile aligns with the typical age group affected by HGSC, yet the concurrent presence of a benign mature teratoma in the opposite ovary introduced diagnostic complexity. Such presentations necessitate a tailored diagnostic approach that combines imaging, cytology, tumor markers, and meticulous histopathology.

## Rationale

Given the rarity of synchronous HGSC and contralateral mature cystic teratoma, reporting this case contributes valuable insight to gynecologic oncology. Very few published cases describe malignant epithelial tumors coexisting with benign germ cell tumors in opposite ovaries, and even fewer include detailed gross, microscopic, immunohistochemical, and clinical correlations. Documenting such cases enhances understanding of ovarian tumor heterogeneity and highlights key diagnostic considerations that may influence surgical planning.

## Aim of the report

The objective of this case report was to:

1. Describe the clinical, radiological, surgical, and pathological features of synchronous high-grade serous carcinoma and contralateral mature cystic teratoma;
2. Discuss diagnostic challenges, differential diagnoses, and implications for management and follow-up.

By presenting this case, we aim to support the clinicians in recognizing rare dual-origin ovarian pathologies and reinforce the critical role of multidisciplinary assessment in achieving optimal patient outcomes.

## Case history

A 57-year-old postmenopausal woman from India, gravida 3 para 3, presented with a gradual increase in abdominal girth, lower abdominal discomfort, early satiety, and intermittent bloating for 3 months. She denied weight loss or gastrointestinal bleeding. Her medical history was unremarkable, with no known chronic illnesses or previous surgeries. She reported no family history of breast, ovarian, or colorectal cancer and no features suggestive of hereditary cancer syndromes. On general examination, she appeared stable, while abdominal examination revealed a firm, irregular mass arising from the pelvis and extending to the level of the umbilicus. Shifting dullness indicated the presence of ascites. Pelvic examination demonstrated a right adnexal mass with restricted mobility and subtle nodularity in the pouch of Douglas.

Initial laboratory investigations revealed mild anemia with hemoglobin of 10.8 g/dL, while liver and renal function tests were within normal limits. Tumor marker evaluation showed a markedly elevated CA-125 level of 628 U/mL, whereas CEA and CA 19-9 levels were normal. These findings raised suspicion of an epithelial ovarian malignancy. Contrast-enhanced CT of the abdomen and pelvis further delineated the pathology by demonstrating a bulky right ovarian mass with solid papillary projections and areas of necrosis, along with a large left adnexal lesion characterized by fat–fluid levels, a Rokitansky protuberance-like structure, and tooth-like calcifications suggestive of a mature cystic teratoma. Moderate ascites, omental caking, and peritoneal nodularity were noted, and mild endometrial thickening measuring 11 mm was also observed. There was no evidence of hepatic or distant metastasis. Diagnostic paracentesis yielded straw-colored ascitic fluid containing malignant epithelial cells with prominent nucleoli, confirming peritoneal carcinomatosis.

Following multidisciplinary tumor board evaluation, the patient underwent total abdominal hysterectomy with bilateral salpingo-oophorectomy, infracolic omentectomy, and peritoneal biopsies. Complete macroscopic cytoreduction was attempted, although residual peritoneal nodules less than 1 cm remained along the omentum. Gross examination of the surgical specimen revealed that the right ovary measured 3.8 × 2.4 × 2 cm and showed a partially cystic structure with a solid papillary whitish area measuring 1.5 × 0.6 cm. The right fallopian tube measured 5.7 cm and exhibited several serosal nodules. The left ovary was replaced by a large unilocular cystic mass measuring 11.2 × 11 × 8 cm containing sebaceous material, hair shafts, and calcified areas, grossly consistent with a mature cystic teratoma. The omentum displayed multiple nodular deposits of varying size, while the uterus exhibited senile cystic atrophy with a benign endometrial polyp and no myometrial invasion.

Microscopic examination of the right ovary revealed high-grade serous carcinoma, characterized by complex papillary and micropapillary structures, marked nuclear atypia, brisk mitotic activity, and psammoma bodies. Tumor infiltration into the right fallopian tube and cervical stroma was evident, and omental biopsies confirmed metastatic carcinoma. Immunohistochemical staining demonstrated strong, diffuse WT-1 and PAX8 positivity, a mutant p53 overexpression pattern, a high Ki-67 proliferative index of approximately 70%, focal estrogen receptor positivity, and progesterone receptor negativity, supporting the diagnosis of Müllerian-type HGSC. In contrast, microscopic sections from the left ovarian mass revealed well-differentiated ectodermal, mesodermal, and endodermal derivatives without immature or malignant elements, confirming a benign mature cystic teratoma. Based on surgical and pathological findings, the disease was staged as FIGO IIIC (pT3c Nx Mx).

**Figure 1:**
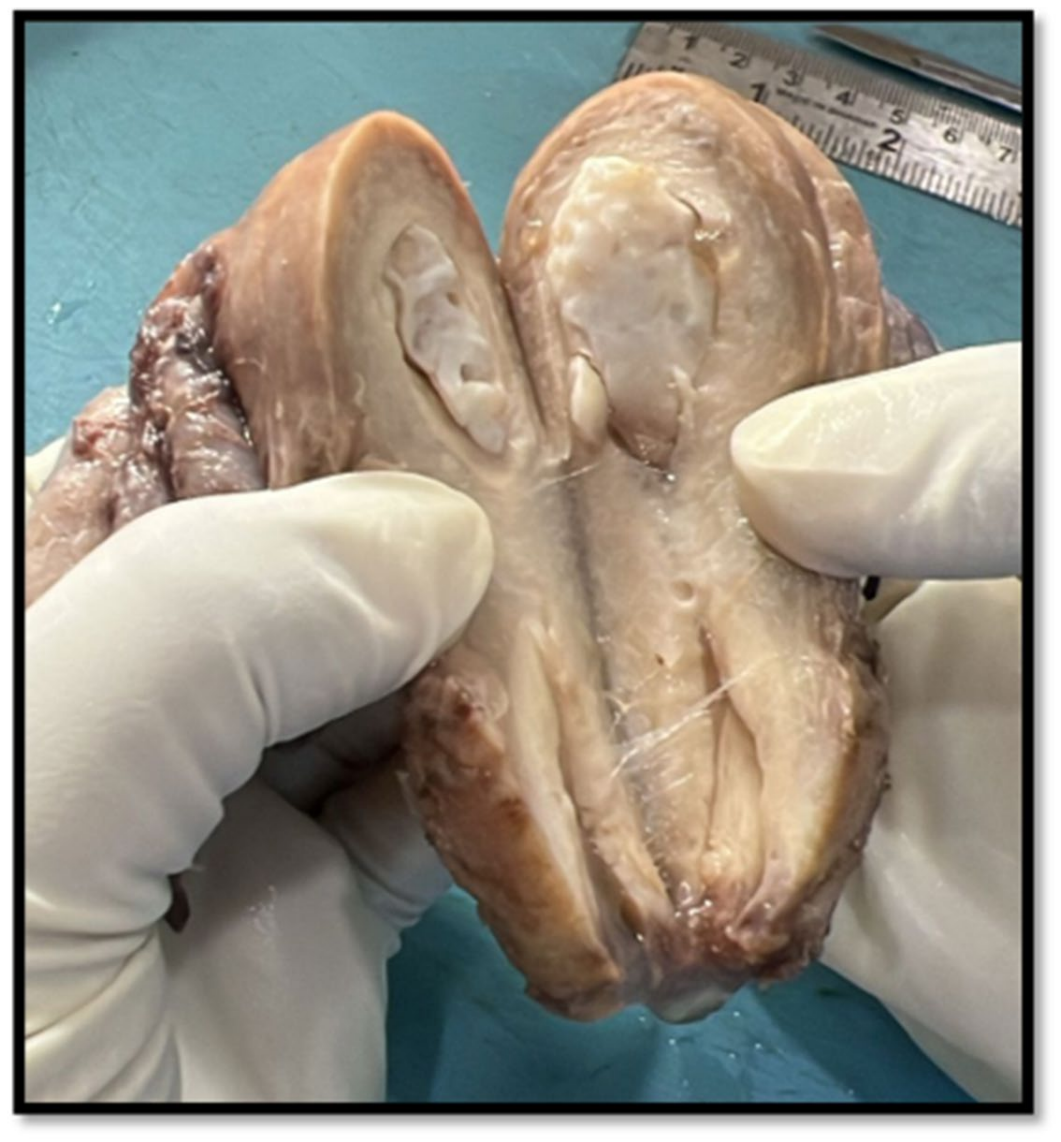
Gross photograph showing a bisected ovary with a unilocular cystic lesion containing pultaceous material and hair shafts, consistent with a mature cystic teratoma (dermoid cyst). The internal surface appears smooth with areas of calcification and sebaceous content. The size of the mass was approximately 9 × 5 × 4 cm.

**Figure 2:**
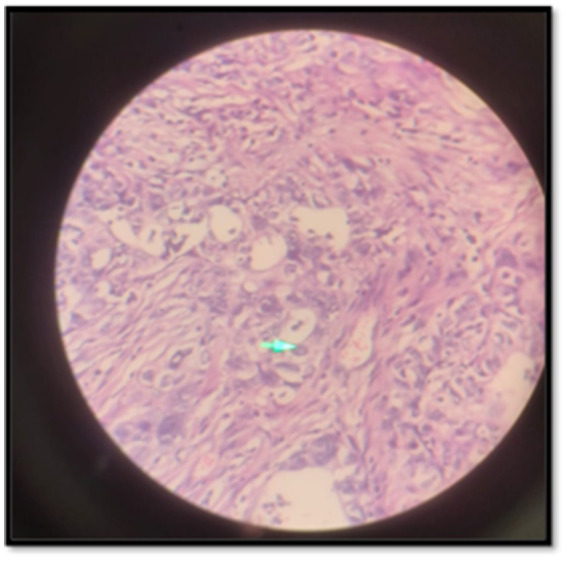
Cystic adenocarcinoma.

**Figure 3:**
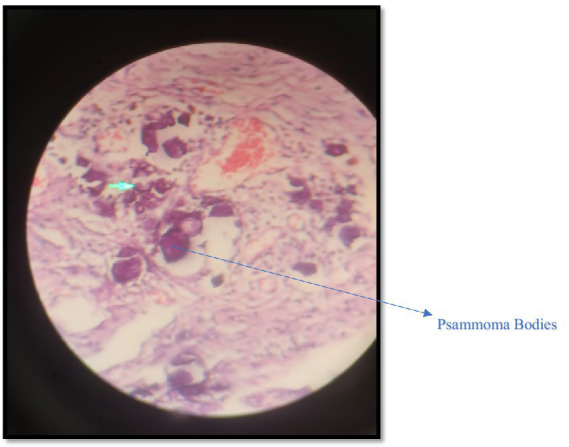
Cystic adenocarcinoma showing psammoma bodies.

Postoperatively, the patient received adjuvant chemotherapy with paclitaxel and carboplatin. After 3 months, her CA-125 level declined to 42 U/mL, and imaging showed no recurrent disease. After 6 months, she remained asymptomatic with no radiological evidence of progression. By 9 months, she reported significant improvement in abdominal comfort and emotional wellbeing, and she tolerated chemotherapy well without major adverse events. From the patient’s perspective, understanding that one tumor was benign and the other malignant provided reassurance, and she expressed satisfaction with the clarity of communication and the outcomes of her treatment (Figures 1–3).

## Possible molecular profiling findings

Considering the diagnosis of HGSC of Müllerian origin and FIGO stage IIIC illness, complete molecular profiling revealed many distinctive molecular changes. The most prevalent and defining aberration in HGSC is a TP53 mutation, which corresponds to the aberrant (mutant-type) p53 immunohistochemical staining pattern observed in this case. Furthermore, approximately 40–50% of HGSCs have a homologous recombination deficit (HRD), usually caused by BRCA1 or BRCA2 mutations that may be germline or somatic. Testing for BRCA mutations is therapeutically relevant, as it predicts a better response to platinum-based chemotherapy and eligibility for PARP inhibitor maintenance treatment.

Other probable molecular discoveries include alterations in RAD51C, RAD51D, PALB2, and ATM, all of which contribute to HRD. HGSC is distinguished from low-grade serous carcinoma by its genomic instability and extensive copy number variations, as well as the absence of activating mutations in KRAS, BRAF, or PTEN. Molecular profiling may also identify CCNE1 amplification, which is associated with platinum resistance and adverse prognosis. Overall, comprehensive molecular testing in such cases informs prognosis, guides targeted therapy selection, and supports genetic counseling for inherited cancer risk (Table 1).

**Table 1:** CARE-compliant timeline.

Time point	Clinical events and findings
Three months before presentation	Progressive abdominal distension, lower abdominal discomfort, early satiety, and bloating. No weight loss or systemic symptoms
Initial presentation	Physical examination revealed a firm pelvic mass with ascites. No significant past medical history or family history of ovarian or breast malignancy
Day 0 (first hospital visit)	Baseline laboratory investigations showed mild anemia (Hb of 10.8 g/dL) with normal renal and liver function tests. Tumor markers revealed markedly elevated CA-125 (628 U/mL), while CEA and CA 19–9 levels were within normal limits
Day 4	Contrast-enhanced CT (CECT) abdomen–pelvis demonstrated a right ovarian solid–cystic mass with papillary projections, left adnexal fat–fluid lesion suggestive of dermoid cyst, ascites, and omental caking
Day 5	Diagnostic ascitic fluid aspiration performed; cytology positive for malignant epithelial cells. Multidisciplinary tumor board discussion undertaken
Day 10	Patient underwent total abdominal hysterectomy with bilateral salpingo-oophorectomy, infracolic omentectomy, and peritoneal biopsies
Postoperative days 1–5	Uneventful postoperative recovery; patient discharged on postoperative Day 5
Week 2 (histopathology report)	Right ovary: high-grade serous carcinoma with tubal and cervical stromal invasion; immunohistochemistry positive for WT-1 and PAX8, p53 mutant pattern, Ki-67 ~ 70%. Left ovary: benign mature cystic teratoma. Omental deposits positive for metastasis. Final staging: FIGO II

## Discussion

The synchronous occurrence of HGSC in one ovary and a mature cystic teratoma in the contralateral ovary is rare, with only a few similar cases documented. Although mature cystic teratoma is the most common ovarian germ cell tumor, the most common overall ovarian neoplasm is serous cystadenoma, whereas HGSC remains the leading epithelial malignancy in postmenopausal women. The present case is noteworthy because the two ovarian lesions differ entirely in their histogenesis, morphology, and biologic behavior, making this presentation distinct from the more commonly encountered bilateral epithelial or bilateral germ cell tumors.

The rarity of this case becomes more evident when compared with existing literature. Chahkandi et al. (5) reported a mature teratoma with coexisting mucinous cystadenocarcinoma within the same ovary, representing a collision tumor rather than two anatomically separate lesions. Their findings differ from this study because the tumors were intermixed, whereas in the present case, the carcinomatous and teratomatous components were confined to opposite ovaries, supporting the hypothesis of two independent neoplastic processes. Similarly, Matalliotakis et al. (6), in a large retrospective review, identified associations between teratomas and benign serous cystadenomas in only 4.9% of patients and reported no cases associated with serous cystadenocarcinoma, underscoring the exceptional nature of our finding.

Grisales-Gallo et al. (7) described a pediatric case involving a teratoma coexisting with a giant serous cystadenoma, a benign combination that nevertheless contributed to diagnostic difficulty. Their report, although involving benign pathology, supports the notion that mixed ovarian tumors—whether benign or malignant—can complicate preoperative assessment. In another relevant report, Kumar et al. (8) documented bilateral ovarian tumors of differing histologies, including cystadenofibroma with contralateral collision lesions, demonstrating the heterogeneity of ovarian tumor biology and the need for extensive sampling in all adnexal masses. Our case reinforces this principle, particularly since HGSC may involve both ovaries in advanced disease, making comprehensive histopathological examination essential to avoid misdiagnosis.

Pant et al. (9) reported mature cystic teratomas with unusual coexisting neoplasms, emphasizing the necessity of thorough sampling because unexpected or occult lesions may be present. Their observation aligns with our approach, which confirmed that the teratoma in this case contained only benign mature elements with no signs of malignant transformation. Additional comparison may be drawn with Abduljabbar et al. (10), who described malignant degeneration of a mature teratoma accompanied by a serous cystadenoma, though not HGSC, suggesting that combinations of germ cell and epithelial neoplasms, while uncommon, can occur in varied patterns. Unlike their case, our patient had a high-grade malignancy on one side and a clearly benign tumor on the other, strengthening the interpretation of independent tumorigenesis.

Preoperative imaging was critical in characterizing the bilateral ovarian tumors and ruling out other possible diagnoses. Ultrasonography and contrast-enhanced CT revealed distinctly different morphologies in each ovary (11). The malignant lesion revealed a complicated solid-cystic mass with irregular septations, papillary projections, and post-contrast enhancement, which are characteristic of serous cystadenocarcinoma and correspond to considerably raised serum CA-125 values (12). The contralateral ovarian mass showed fat-density regions, calcifications, and a well-defined cystic component, all of which are indicative of a benign mature cystic teratoma. The lack of omental caking, peritoneal deposits, substantial ascites, or distant metastasis reduced the risk of metastatic disease (13). Mucinous tumors were thought to be rare since they lacked multiloculated cysts and had the distinctive “stained glass” appearance (12). Thus, imaging data, confirmed by tumor marker association, suggested synchronous but histologically separate ovarian tumors rather than metastatic or mucinous disease (13).

The immunohistochemical profile in this case, characterized by WT-1 and PAX8 positivity, mutant-pattern p53 expression, and a high Ki-67 index, is consistent with HGSC and corroborates findings from similar reports. The presence of cervical and fallopian tube involvement further reinforces the Müllerian origin suggested by IHC and aligns with the current understanding that many HGSCs arise from serous tubal intraepithelial carcinoma (STIC). These features distinguish the malignancy from rare malignant transformations of teratoma, which more commonly result in squamous cell carcinoma.

## Differential diagnosis based on the imagining findings

Several alternative diagnoses for the bilateral ovarian tumors were evaluated based on the imaging results. The complicated solid–cystic lesion with irregular septations, papillary projections, and contrast enhancement suggested the presence of additional primary epithelial ovarian cancers, such as endometrioid or clear cell carcinoma. However, serous cystadenocarcinoma outperformed these subtypes because of its conspicuous papillary architecture, imaging aggressiveness, and increased CA-125 values (11). Borderline ovarian tumors were also evaluated since they can have papillary projections, although the presence of solid enhancing components and invasive characteristics on imaging reduced the likelihood of a borderline lesion (13).

Given the bilateral form of the masses, metastatic ovarian involvement, particularly Krukenberg tumor, was a relevant differential diagnosis. This was considered improbable because there was no known underlying malignancy, no predominantly solid bilateral masses, and no indications of peritoneal deposits, omental caking, or distant metastases (12). Mucinous ovarian tumors were rejected due to the lack of large multiloculated cysts with thin septa and the distinctive “stained glass” appearance on imaging. Functional hemorrhagic cysts and endometriomas were considered improbable, given that these lesions do not normally have enhanced solid components or papillary projections. The contralateral lesion’s fat accumulation, calcifications, and well-defined cystic shape were more indicative of a benign mature cystic teratoma than a developing teratoma or malignant change (14).

## Practice-challenging insights

This study demonstrates various practice-challenging elements of gynecologic oncology. First, the coexistence of a malignant epithelial ovarian tumor and a contralateral benign mature cystic teratoma might be diagnostically deceptive on imaging, possibly delaying the detection of advanced malignancy. Careful radiologic–pathologic correlation is critical, especially in postmenopausal women, for whom any adnexal tumor raises serious concerns.

Second, the occurrence of advanced-stage HGSC with tubal and cervical stromal invasion highlights the growing concept of the fallopian tube as the site of genesis for many HGSCs, emphasizing the importance of thorough pathological assessment of the tubes (SEE-FIM procedure). Third, while optimal cytoreductive surgery followed by platinum-based chemotherapy remains the standard, the example highlights the need for early molecular profiling (BRCA/HRD testing), which may impact maintenance therapy decisions even in patients who demonstrate a strong initial response. Finally, short-term disease control at 9 months should not obscure the high risk of late recurrence in FIGO IIIC HGSC, highlighting the importance of long-term surveillance and consideration of maintenance strategies.

## Clinical decision-making framework

The patient’s presentation with postmenopausal abdominal distension, ascites, and a complex adnexal mass raised a high suspicion for ovarian malignancy. Initial imaging demonstrated a solid–cystic right ovarian mass with peritoneal involvement, while the contralateral fat–fluid level suggested a benign dermoid cyst, supporting the likelihood of synchronous but biologically distinct lesions. Markedly elevated CA-125 levels and malignant epithelial cells on ascitic cytology further reinforced the diagnosis of advanced epithelial ovarian carcinoma.

Given the evidence of resectable disease and the patient’s clinical fitness, primary cytoreductive surgery was chosen instead of neoadjuvant chemotherapy to achieve optimal tumor debulking and accurate staging.

Comprehensive surgical management, including hysterectomy, bilateral salpingo-oophorectomy, and omentectomy, allowed definitive histopathological diagnosis and staging.

**Figure 4:**
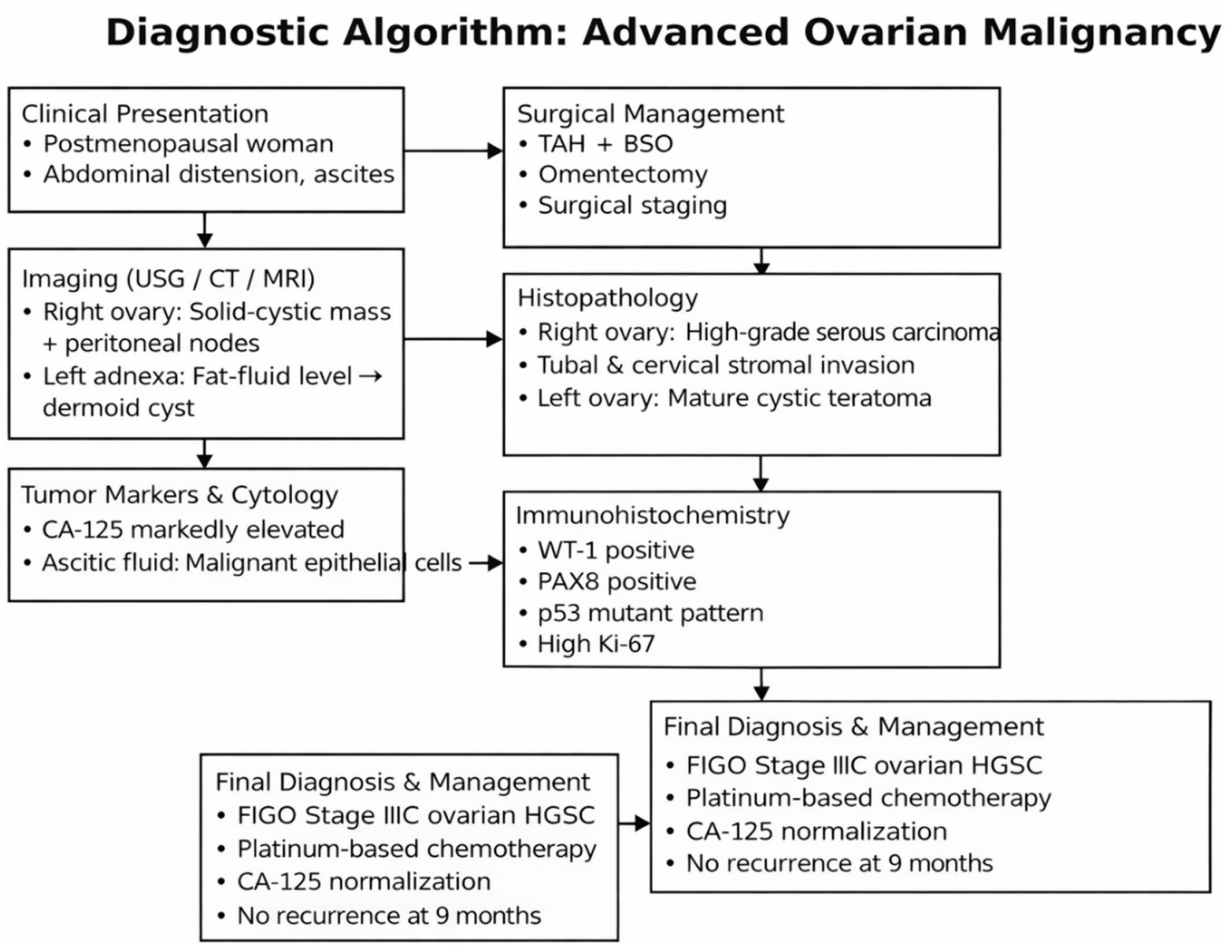
Diagnostic algorithm for the present case (suspected advanced ovarian malignancy).

Histology and immunohistochemistry confirmed high-grade serous carcinoma of Müllerian origin (WT-1+, PAX8+, p53 mutant, and high Ki-67), justifying FIGO stage IIIC classification and the need for adjuvant platinum-based chemotherapy. Postoperative chemotherapy was selected in accordance with standard guidelines for advanced ovarian cancer, resulting in biochemical response, clinical improvement, and no evidence of recurrence at nine-month follow-up (Figure 4).

## Management strategies

To achieve optimal oncologic results while minimizing excessive morbidity, patients with discordant ovarian tumors must be managed in a highly individualized, interdisciplinary manner. Benign mature cystic teratomas are typically treated with cystectomy or unilateral oophorectomy, especially in younger or fertility-seeking patients, whereas HGSC requires extensive surgical staging and maximal cytoreductive surgery, followed by platinum-based chemotherapy. Failure to appropriately categorize these lesions before or during surgery may result in overtreatment of a benign ovary or improper management of an aggressive cancer. Early referral to a gynecologic oncologist is therefore critical for effective surgical planning. Intraoperative frozen-section evaluation can provide significant real-time diagnostic information, particularly when imaging results are contradictory or confusing.

Furthermore, extensive gross and histological sampling of both ovaries is required, since HGSC frequently occurs bilaterally, and mature teratomas, while normally benign, may occasionally undergo malignant change. This rigorous approach ensures accurate diagnosis, proper staging, and customized postoperative therapy, thereby improving prognosis and reducing treatment-related complications.

## Patient perspective

The patient’s stomach pain and distension worsened in the months leading up to the diagnosis, causing her to become increasingly anxious and concerned about her health. Receiving a precise explanation of her condition gave her a sense of comfort, especially when she realized that the tumors in her ovaries were distinct and only one was cancerous. Although the diagnosis of ovarian cancer was emotionally overwhelming, she was reassured by the detailed explanation of the treatment plan and valued the opportunity to participate actively in decision-making. The thought of chemotherapy was initially worrisome owing to concerns about side effects and long-term results, and she experienced panic, exhaustion, and emotional fragility during treatment. However, regular contact and assistance from the healthcare team helped her overcome these obstacles. She experienced considerable improvement in her stomach discomfort and daily functioning following surgery and chemotherapy. While follow-up visits caused some concern, she regarded them as necessary for reassurance and remained optimistic about her recovery, expressing gratitude for the care and support she received.

## Strength and limitations

This report highlights include a full clinicopathological correlation, as well as extensive gross, microscopic, and immunohistochemical evaluations that demonstrate the presence of two synchronous ovarian tumors with completely separate histogenesis. Clear documentation of different radiologic features, intraoperative findings, and histological traits improves diagnostic clarity and allows for appropriate categorization. The inclusion of tumor marker correlation with response to conventional therapy enhances the clinical significance of this case. Furthermore, the rare occurrence of serous cystadenocarcinoma concomitant with a benign mature cystic teratoma offers uniqueness and instructional value to the current literature.

However, this report has certain drawbacks. The nine-month follow-up period reduces the ability to examine long-term oncological outcomes, recurrence risk, and survival. The absence of molecular and genetic profiling limits the investigation of potential common pathogenetic pathways or clonal links between tumors. Because this is a single-case study, the findings are not generalizable and cannot guide standardized management techniques. Furthermore, imaging interpretation may vary among observers, and frozen-section correlation was restricted. Despite these constraints, the case emphasizes the significance of carefully assessing bilateral adnexal masses with discordant characteristics.

## Conclusion

This case demonstrates the unusual occurrence of synchronous high-grade serous carcinoma of one ovary and a contralateral benign mature cystic teratoma, as well as the diagnostic complications posed by discordant bilateral ovarian tumors. Accurate diagnosis is dependent on rigorous clinicoradiological correlation, substantial sampling, and immunohistochemistry confirmation to establish unique histogenesis and staging. Recognition of such presentations is critical to avoiding mismanagement, since treatment techniques differ significantly between malignant and benign ovarian tumors. Early gynecologic oncology involvement and individualized care are critical for best results. This study contributes to the limited literature on synchronous ovarian tumors and emphasizes the significance of thorough evaluation and individualized follow-up in similar cases.

## Data Availability

The original contributions presented in the study are included in the article/supplementary material, further inquiries can be directed to the corresponding author.
